# A metagenome-wide association study of gut microbiota in asthma in UK adults

**DOI:** 10.1186/s12866-018-1257-x

**Published:** 2018-09-12

**Authors:** Qi Wang, Fei Li, Bishan Liang, Yuhu Liang, Sijie Chen, Xiaodong Mo, Yanmei Ju, Hui Zhao, Huijue Jia, Timothy D. Spector, Hailiang Xie, Ruijin Guo

**Affiliations:** 1 BGI Education Center, University of Chinese Academy of Sciences, Shenzhen, 518083 China; 2 BGI-Shenzhen, Shenzhen, 518083 China; 3 China National Genebank, BGI-Shenzhen, Shenzhen, 518083 China; 4 Macau University of Science and Technology, Taipa, Macau, 999078 China; 5 Department of Twin Research and Genetic Epidemiology, King’s College London, London, SE1 7EH UK; 6 Department of Oncology, Nanfang Hospital, Southern Medical University, Guangzhou, Guangdong 510515 People’s Republic of China; 7 Shenzhen Key Laboratory of Human Commensal Microorganisms and Health Research, BGI-Shenzhen, Shenzhen, 518083 China

**Keywords:** Asthma, MWAS, *Eggerthella lenta*, *Faecalibacterium prausnitzii*, SCFAs

## Abstract

**Background:**

Asthma, one of the most common chronic respiratory disorders, is associated with the hyper-activation of the T-cell subset of adaptive immunity. The gut microbiota may be involved in the development of asthma through the production of short-chain fatty acids (SCFAs), exhibiting modulatory effects on Th. So, we performed a metagenome-wide association study (MWAS) of the fecal microbiota from individuals with asthma and healthy controls. And that was the first case to resolve the relationship between asthma and microbiome among UK adults.

**Results:**

The microbiota of the individuals with asthma consisted of fewer microbial entities than the microbiota of healthy individuals. *Faecalibacterium prausnitzii*, *Sutterella wadsworthensis* and *Bacteroides stercoris* were depleted in cases, whereas *Clostridiums* with *Eggerthella lenta* were over-represented in individuals with asthma. Functional analysis shows that the SCFAs might be altered in the microbiota of asthma patients.

**Conclusion:**

In all, the adult human gut microbiome of asthma patients is clearly different from healthy controls. The functional and taxa results showed that the change of asthma patients might related to SCFAs.

**Electronic supplementary material:**

The online version of this article (10.1186/s12866-018-1257-x) contains supplementary material, which is available to authorized users.

## Background

Asthma is a prevalent chronic respiratory disorder, affecting millions of people globally [1]. Besides several risk alleles associated with asthma have been reported [2], the gut microbiota also acts an essential role in the emergence and development of asthma [3].

The gut microbiota influences metabolic and immune homeostasis [4, 5]. This symbiotic relationship is related to diverse host physiological functions [6]. It is conventionally believed that asthma is associated with the over-activation of the T-cell subset arm of adaptive immunity characterized as the up-regulation of the pro-inflammatory T-cell subset activity [3, 7]. And the microbiome possibly influences the T-cell populations through the microbe-derived short-chain fatty acids (SCFAs), which can regulate T cell activity and further affect asthma [8]. As the metabolic balance of SCFAs depends on the symbiosis of microbiota, an impaired gut micro-ecosystem might be involved in the development of asthma [9]. This idea is supported by the emerging evidence from the study on the microbiota of neonates who subsequently develop asthma during childhood [10], and yet, little is known on association between gut microbiota and asthma in adults.

Here, we present the results of the analysis on the metagenomic data of 36 asthma patients and 185 healthy controls selected from a former study [11]. We identify meta-genomic species (MGSs) [12] characteristic of asthma, and the differentially enriched Gut metabolic modules (GMMs) [13] between asthma and control samples, which revealed an association of altered short chain fatty acid metabolism with asthma.

## Results

### Global alterations of the gut microbiome in asthma

**Fig. 1:**
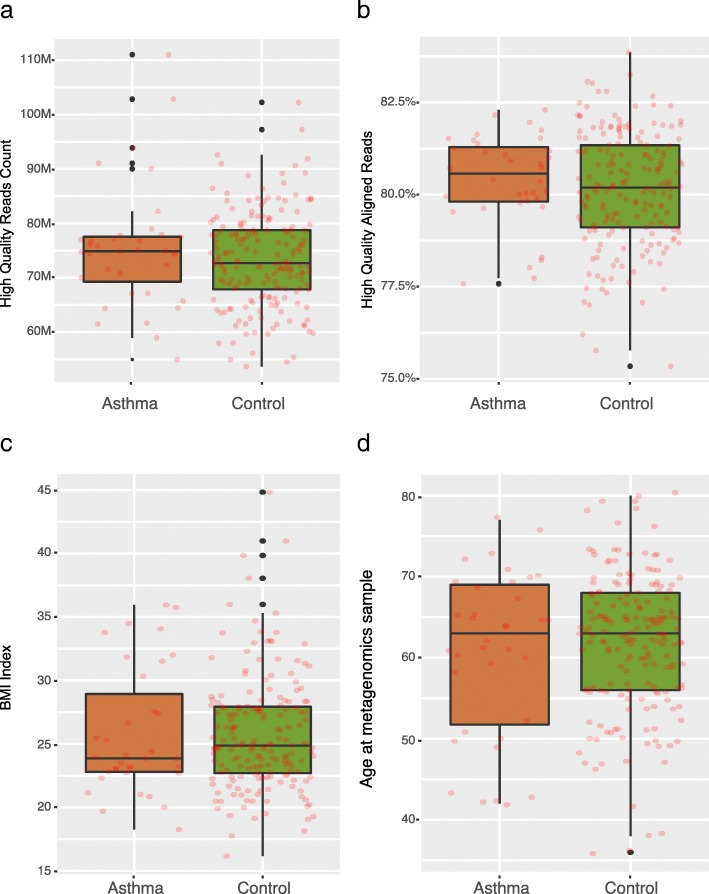
High quality reads and lifestyle factor. **a**-**b** The count (*p* = 0.5096, two-tailed Wilcoxon-rank sum test) and rate of aligned to geneset (*p* = 0.3896, two-tailed Wilcoxon-rank sum test) of high quality reads between asthma and control. **c**-**d** The BMI index (*p* = 0.6609, two-tailed Wilcoxon-rank sum test) and sample age (*p* = 0.715, two-tailed Wilcoxon-rank sum test) of asthma and control

To investigate the gut microbiota in asthma, we extracted the high-quality meta-genomic shotgun sequencing data of 36 asthma cases and 185 control samples from the database of a previous study [11] (Additional file 1: Table S1). These reads were aligned to the human gut microbial gene catalog composing of 11.4 million genes constructed in the same study [11], that leads to the mapping of averaged 80.19% of sequencing reads per sample (Fig. 1a, b, Additional file 1: Table S1B).

To evaluate the potential effects of the clinical and lifestyle factors [14] on the gut microbiota (Fig. 1c, d, Additional file 1: Table S1A), we conducted permutational multivariate analysis of variance (PERMANOVA) [15] with the microbial gene abundance profiles. Patients diagnosed with asthma was associated with an alteration of the global microbiota composition (Additional file 2: Table S2), along with a couple of lifestyle factors, Body Mass Index (BMI)and ages, which show great difference in PERMANOVA (Additional file 2: Table S2), but not significant between asthma and control samples (two-tailed Wilcoxon-rank sum test, *p* > 0.5) (Fig. 1c, d).

**Fig. 2:**
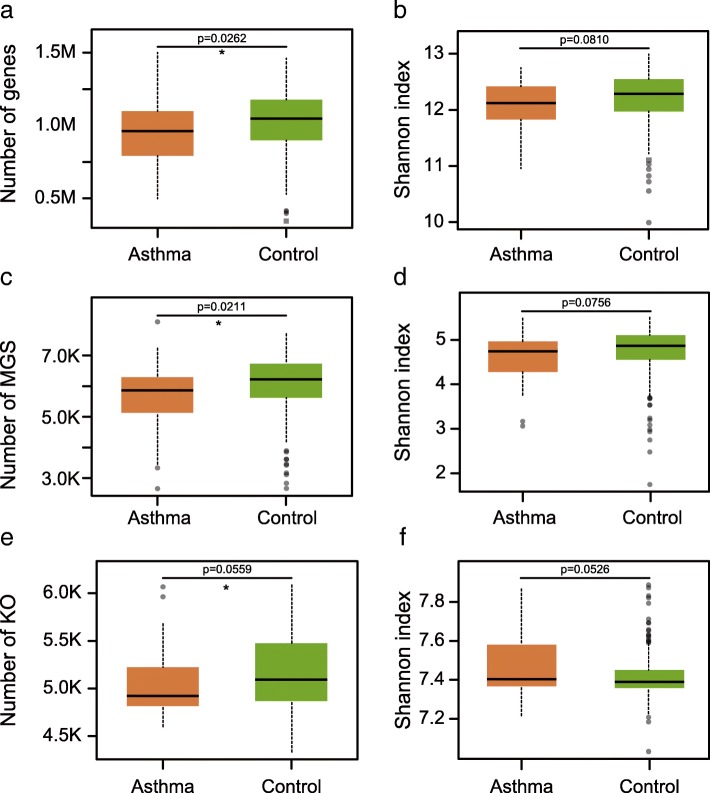
Reduced gut microbial richness in asthma. (**a**-**f**) Richness and alpha-diversity (Shannon index) at the gene, MGS and KO level of the two cohorts (Test by two-tailed Wilcoxon-rank sum test). Box plots showing both the richness values or diversity values and their density

Gut microbial richness and evenness were compared between control and asthma samples at the level of genes, MGSs and KEGG Orthology (KOs) (Fig. 2). The numbers of genes, MGSs (*p* = 0.026, *p* = 0.021, respectively, two-tailed Wilcoxon-rank sum test, Fig. 2a, c), and KOs (*p* = 0.0559, Welch’s t-test, Fig. 2e) are lower in asthma patients compared to control, while the alpha-diversities at the three levels show difference between these two groups (Fig. 2b, d, f).

To show the differences of main genera and phyla level abundance between asthma patients and healthy controls, we selected 117 genera and 15 phyla which occurrence rate more 50% versus patients and control. Then we calculated the mean relative abundance of selected genera and phyla then compared the top 15 genera and top 5 phyla versus patients and healthy people (Additional file 3: Figure S1, Additional file 4: Figure S2, Additional file 5: Table S3, Additional file 6: Table S4). In the genera level the *Faecalibacterium* is significant enriched in control, *Blautia* major in asthma patents (two-tailed Wilcoxon-rank sum test, *p* < 0.05, Additional file 3: Figure S1, Additional file 5: Table S3).

And to clear up the potential bias of twins, we just picked data from only one of the twins at random for each pair, that also showed imbalance between these two cohorts (Additional file 7: Figure S4).

These results demonstrate a reduction of gut microbial content under the condition of asthma (especially in gene and MGS level), indicating the undergrowth of certain microbial entities in patients with asthma.

### MGSs characteristic of asthma

**Fig. 3:**
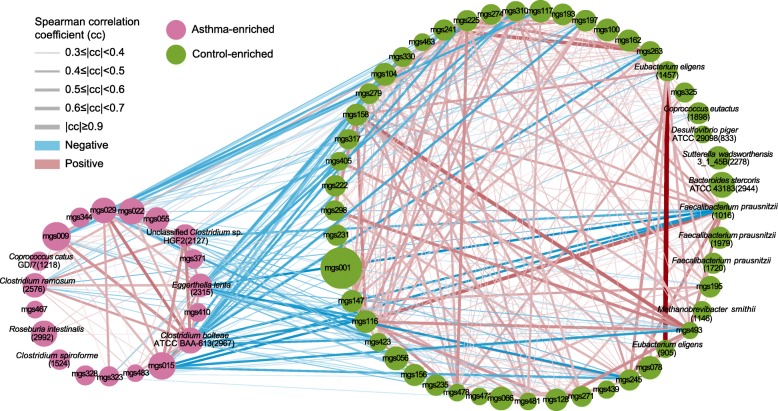
Differentially enriched MGS, Control versus asthma: For all MGSs with more than 700 genes, the orientation of enrichment was determined by two-tailed Wilcoxon-rank sum test ((U statistic of two-tail Wilcoxon rank-sum test, *P* < 0.05, FDR < 0.2, Effect size equal to ($1-\frac{2U}{nm}\Big)$, Additional file 5: Table S3). Size of the nodes consistent with the number of genes (700~ 3645) in the MGSs. MGSs annotated to species were colored according to enrichment. Edges between nodes indicated Spearman’s correlation > 0.3 (red) or < − 0.3 (blue), calculated according to the samples under comparison

To obtain more insights into the signatures of the gut microbiome in asthma or healthy samples, co-abundance gene present in more than 90% samples were clustered into 12,226 co-abundance gene groups (CAGs), including 500 meta-genomic species (MGSs) which have no fewer than 700 genes and represent microbial species [16]. A total of 68 of the MGSs differ significantly in abundance between asthma and control samples (two-tailed Wilcoxon-rank sum test, *p* < 0.05, FDR < 0.2), with 19 of these being more abundant in asthma samples (Fig. 3, Additional file 8: Table S5). Correlation network was constructed for MGS enriched in either group (Fig. 3).

*Faecalibacterium prausnitzii* is a butyrate-producing bacterium, same as *Coprococcus eutactus* [16], and also an anti-inflammatory commensal bacterium [17]. We found *Faecalibacterium prausnitzii* is enriched in the gut of healthy people, and this finding is consisted with former studies that *F. prausnitzii* was also enriched in healthy infants but not in asthma development infants [10]. (Fig. 3, Additional file 8: Table S5). Moreover, the species, such as *Sutterella wadsworthensis* and *Bacteroides stercoris*, are found to be enriched in our control, this finding is identical with the former 16S study, in which *S.wadsworthensis* and *B.stercoris* were also found to be enriched in healthy people [18, 19] (Fig. 3, Additional file 8: Table S5). The abundance of Eubacterium eligens, a bacterium previously associated with pectin fermentation in the colon is higher in control samples than in asthma samples [20] (Fig. 3, Additional file 8: Table S5). The abundance of *Methanobrevi*bacter smithii, which makes up 10% of all anaerobes in the colons of healthy adults and that can be a therapeutic target for reducing energy harvest in obese humans [21], is higher in control (Fig. 3, Additional file 8: Table S5).

In contrast, the asthma enriched *Clostridiums* include *Clostridium bolteae, Clostridium ramosum,* and *Clostridium spiroforme.* The first species is targeted for treat and diagnostic for autism [22]. *Clostridium ramosum,* associated to obesity and the down-regulation of it can reduce the severity of high-fat diet-induced obesity [23]. Also, *Clostridium spiroforme* is higher in asthma than in control samples (Fig. 3, Additional file 8: Table S5). The asthma-enriched species *Eggerthella lenta* is identified in inflammatory bowel disease patients [24] (Fig. 3, Additional file 8: Table S5).

Besides the abundance differences between asthma and control samples, the MGSs also show differences in network structure (Spearman’s correlation coefficient (cc) ≥ 0.3 or ≤ − 0.3, Fig. 3). Most notably, the asthma-enriched species *Clostridium bolteae and Eggerthella lenta* has negative correlations with the asthma-depleted species *Faecalibacterium prausnitzii* (Fig. 3, Additional file 8: Table S5)*.* These results demonstrate imbalances in the composition and inter-species relationship in the gut microbiome of asthma patients as compared to healthy controls.

### Functional changes in the microbiome of asthma

Here, we used the GMMs [13] to investigate the microbial functional difference between the asthma patients and healthy control (Table.1; Additional file 9: Table S6*,* two-tailed Wilcoxon-rank sum test*, P < 0.05, FDR. = 0.2)*. Results show that the models related to the metabolism of several amino acids, glycometabolism, lipid metabolism are enriched in the asthma patients. On the contrary, the modules enriched in the healthy people are related to hydrogen metabolism, butyrate production, aspartate degradation, acetate production and pyruvate processing (Table.1, Additional file 9: Table S6). These changes indicate deviations from the normal nutrient metabolic states in the microbiota of asthma patients, especially those in SCFA metabolism which enriched in control samples has modulatory effects on Th2 [8]. Meanwhile according to the virulence factor database (VFDB), more virulence factors significantly enriched in the asthma samples compared to the control samples (Additional file 10: Figure S3, Additional file 11: Table S7).

**Table 1:** Different GMMs be enriched: This table list the statistically significant GMMs (U statistic of two-tail Wilcoxon rank-sum test, *P* < 0.05, FDR < 0.26, Effect size equal to ($1-\frac{2U}{nm}\Big)$), where n and m is the sample size of case and control)); whole GMMs please refer to Additional file 9: Table S6

GMM_ID	Functional assignment	*P* value	Effect size
Asthma enriched			
MF0074	pyruvate: formate lyase	0.000162909	0.397597598
MF0085	urea degradation	0.00026136	0.384984985
MF0029	aspartate degradation II	0.000862902	0.351351351
MF0063	glyoxylate bypass	0.003920233	0.304204204
MF0049	threonine degradation I	0.004991377	0.296096096
MF0040	proline degradation	0.008443348	0.277777778
MF0091	ethanol production II	0.0093327	0.274174174
MF0021	xylose degradation	0.011645774	0.266066066
MF0051	arginine degradation I	0.012930992	0.262162162
MF0059	anaerobic fatty acid beta-oxidation	0.020317732	0.244744745
MF0050	threonine degradation II	0.030266319	0.228528529
MF0069	NADH: ferredoxin oxidoreductase	0.035386145	0.221921922
Control enriched			
MF0098	hydrogen metabolism	0.002663119	−0.316816817
MF0088	butyrate production I	0.017167351	−0.251351351
MF0028	aspartate degradation I	0.028985702	−0.23033033
MF0086	acetyl-CoA to acetate	0.029620059	−0.229429429
MF0073	pyruvate: ferredoxin oxidoreductase	0.036904556	−0.22012012

## Discussion

Herein we aligned our high-quality reads to the lasted human gut microbial gene catalog [12] and demonstrated the difference of identified genes, phylogenies, and functions in the gut microbiome of asthma patients and healthy controls through the metagenome-wide association study.

Some enriched species were also reported in previous study [16–23, 25] and the SCFAs which was depleted in case revealed some functional changes in asthma patients. To explore more in this field, more analysis should be conducted in future researches.

It was believed that asthma is associated with the over-activation of the T-cell subset of adaptive immunity characterized by the up-regulation of the pro-inflammatory T-cell subset activity [8]. Meanwhile, the SCFA metabolism in our gut could regulate the activity of T-cell subset activity as well [8, 26]. And the functional and taxa results shows that not just the SCFAs metabolism, moreover some SCFAs-producing species, *F. prausnitzii* [16, 17] and *C.eutactus* [16]*,*also depleted in asthma patients. The alteration possibly modulating both the innate and the adaptive immune system might play an important role in the asthma pathogenesis.

## Conclusions

All in all, the human gut microbiome of asthma patients is clearly different from healthy controls. The lack of diversity of the gut microbiome is presented in asthma patients. And the absence of certain bacteria related to SCFA metabolism in asthma patient might accelerate the emergence and development of asthma. The asthma-depleted species, *F. prausnitzii* and *C.eutactus* were butyrate-producing bacteria. It is anticipated that exploring the gut microbial communities in this study will contribute to finding more treatments for asthma and provide a brand-new perspective of human–microbial relationships.

## Methods

### Aim and design of the study

We used the MWAS [24] methods to dig into the difference between asthma patients and healthy and found the potential treatment for asthma in the level of metagenomics.

### Material

The sequencing data of 221 sample, including 36 asthma patients and 185 healthy controls were from the former study [12]. The reads aligned to the updated 11.4 million genes catalog [12] after filtered low quality reads and the reads align to host genome(hg19) with in-house scripts.

### Taxonomic annotation and abundance calculation

To assign the taxa of target genes, we used the database, Integrated Microbial Genomes (IMG, v400), with the inner pipeline detailed previously [12], the abundance of taxa was calculated by the sum of corresponding genes.

### Alpha-diversity and gene count

The within-sample diversity was calculated by gene profile of samples with Shannon index, as described previously [12]. Genes were considered present with more than one read map to it.

### Remove the bias of twins

To eliminate the bias from twins, we randomly selected the one from the pairs with the ‘random’ function in Python (Python 2.7).

### PERMANOVA of the effects of related factors

To evaluate the effects of the clinical and lifestyle factors on listed, we calculated the Permutational multivariate analysis of variance (PERMANOVA) [15] with the gene abundance files of the samples. The Bray-Curtis distance and 9999 permutations in R (3.2.5, vegan package) were used.

### Metagenome-wide association study

To investigate the different taxa of the fecal microbiome between healthy controls and asthma patients. We clustered all genes were presented at least in 90% samples into MGSs according to previous describe [12]. Taxonomic annotation and abundance characteristics of the MGSs were carried out as described previously [12]. When comparing two groups, MGSs were further calculated according to Spearman’s correlation between their abundances in all samples, and the software, Cytoscape 3.4.0, was used to visualize the co-occurrence network. The Wilcoxon rank-sum test (*P* < 0.05) was used to determine the orientation of enrichment.

### Gut metabolic modules analysis

Each GMM abundance was calculated as the median of KO abundance with 66% coverage just as showed in the former article [13]. And When comparing two groups, the Wilcoxon rank-sum test (*P* < 0.05) was used to determine the orientation of enrichment.

### Virulence factors

Virulence factors were analyzed according to VFDB (2585 proteins as of 16 August 2016). Genes in the reference gut microbiome gene catalog were identified as these virulence factors (best match according to BlastP, identity > 35%, score > 60), and their relative abundances could then be determined accordingly and only accept present in more than 50% samples.

## Additional files

Additional file 1: **Table S1.** Sample information: The clinical and sequencing information of the sample. (XLS 71 kb)

Additional file 2: **Table S2.** Factors influence the gut microbiome: Test the factors influence the gut microbiome with PERMANOVA. (XLS 7 kb)

Additional file 3: **Figure S1.** The top 15 genera (the mean relative abundance more than 0.46%) in the cohort versus asthma patients to control individuals (two-tailed Wilcoxon-rank sum test, Additional file 5: Table S3c). Genus in blue and red denote asthma-enriched and control-enriched genus respectively (two-tailed Wilcoxon rank-sum test, *P* < 0.05). We selected 117 genera which occurrence rate more 50% versus patients and control as core genera (two-tailed Wilcoxon rank-sum test, *P* < 0.05, FDR < 0.26). (PDF 302 kb)

Additional file 4: **Figure S2.** The top 5 phyla (the mean relative abundance more than 1.78%) in the cohort between asthma patients and control individuals (two-tailed Wilcoxon-rank sum test, Additional file 6: Table S4c): Phyla in blue and red denote asthma-enriched and control-enriched phyla respectively (two-tailed Wilcoxon rank-sum test, *P* < 0.05). (PDF 133 kb)

Additional file 5: **Table S3.** The genus: The relative abundance and statistical test of the genus. (XLS 1084 kb)

Additional file 6: **Table S4.** The phylum: The relative abundance and statistical test of the phylum. (XLS 95 kb)

Additional file 7: **Figure S4.** Reduced gut microbial richness in only one twin sample. (a-f) Richness and alpha-diversity (Shannon index) at the gene, MGS and KO level of the two cohorts (Test by two-tailed Wilcoxon-rank sum test). Box plots showing both the richness values or diversity values and their density. (PDF 262 kb)

Additional file 8: **Table S5.** MGSs from the cohort: The relative abundance and statistical test of the MGSs. (XLS 1648 kb)

Additional file 9: **Table S6.** Differentially enriched GMMs: The relative abundance and statistical test of the GMMs. (XLS 394 kb)

Additional file 10: **Figure S3.** The number of significant enrichment virulence factor (VF): we counted the significant (two-tailed Wilcoxon rank-sum test, *P* < 0.05) enrichment VF in different cohort. (PDF 92 kb)

Additional file 11: **Table S7.** The VFG: The relative abundance and statistical test of the VFGs. (XLS 3741 kb)

## Abbreviations

BMI: Body Mass Index
CAGs: Co-abundance gene group
GMMs: Gut metabolic modules
KO: KEGG Orthology
MGSs: Meta-genomic species
MWAS: Metagenome-wide association study
PERMANOVA: Permutational multivariate analysis of variance
SCFAs: Short-chain fatty acids
VF: Virulence factor
VFDB: Virulence factor database
