# Supplementary figures and images for: A metagenome-wide association study of gut microbiota in asthma in UK adults
Source: BMC Microbiol. 2018 Sep 12;18:114. doi: 10.1186/s12866-018-1257-x (PMC6134768; doi:10.1186/s12866-018-1257-x)

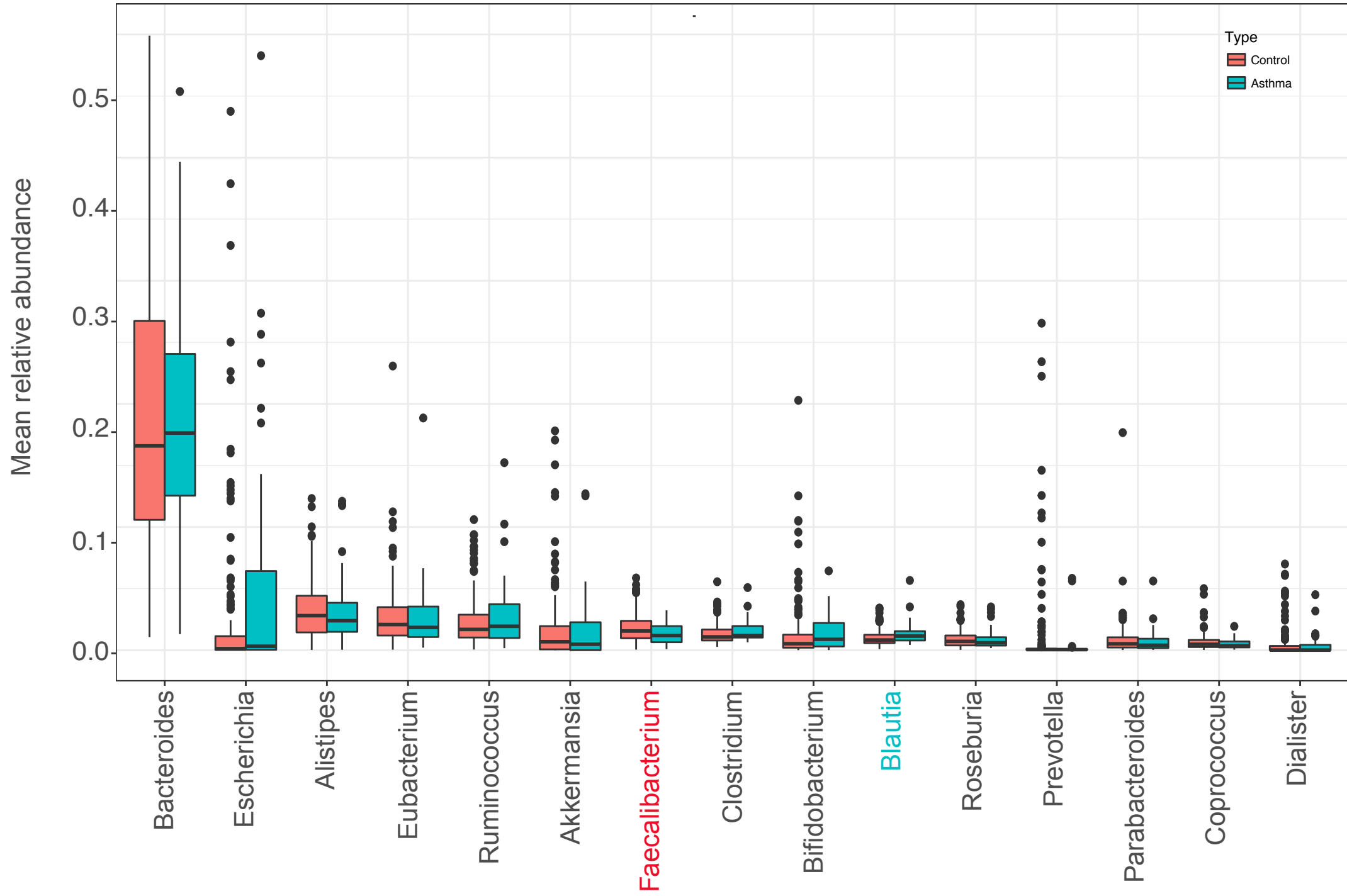

Supplement: Supplementary file 3 — Figure S1. The top 15 genera (the mean relative abundance more than 0.46%) in the cohort versus asthma patients to control individuals (two-tailed Wilcoxon-rank sum test, Additional file 5: Table S3c). Genus in blue and red denote asthma-enriched and control-enriched genus respectively (two-tailed Wilcoxon rank-sum test, P < 0.05). We selected 117 genera which occurrence rate more 50% versus patients and control as core genera (two-tailed Wilcoxon rank-sum test, P < 0.05, FDR < 0.26). (PDF 302 kb) [file 12866_2018_1257_MOESM3_ESM.pdf]

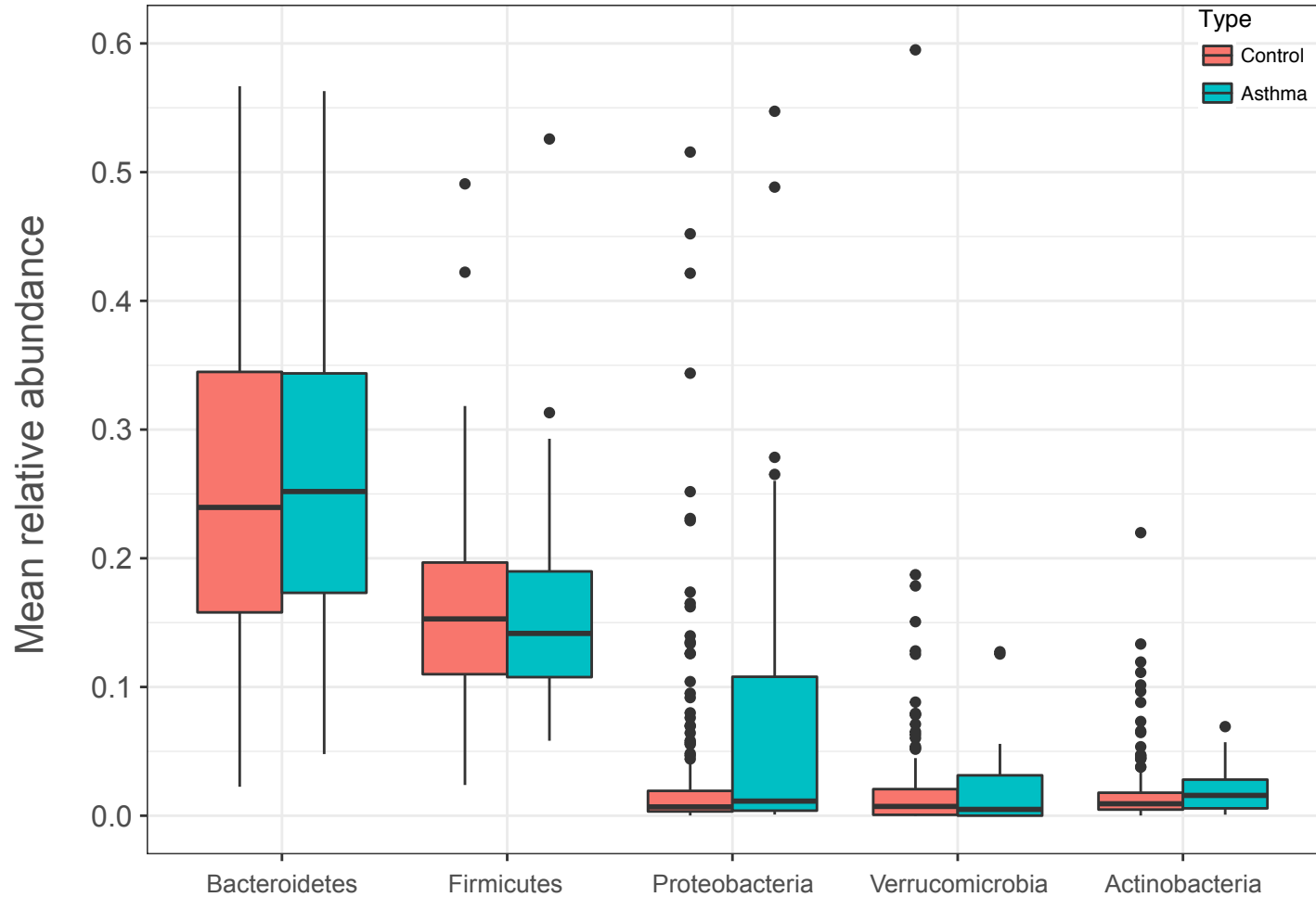

Supplement: Supplementary file 4 — Figure S2. The top 5 phyla (the mean relative abundance more than 1.78%) in the cohort between asthma patients and control individuals (two-tailed Wilcoxon-rank sum test, Additional file 6: Table S4c): Phyla in blue and red denote asthma-enriched and control-enriched phyla respectively (two-tailed Wilcoxon rank-sum test, P < 0.05). (PDF 133 kb) [file 12866_2018_1257_MOESM4_ESM.pdf]

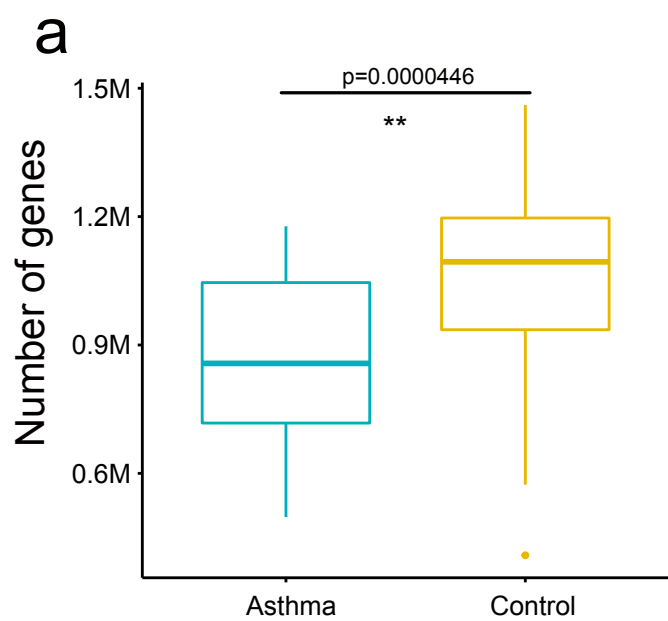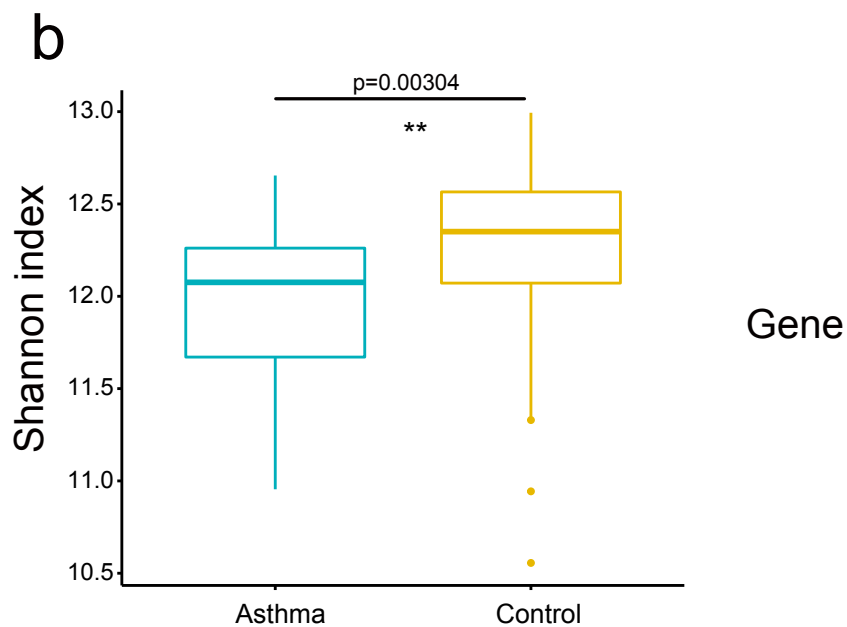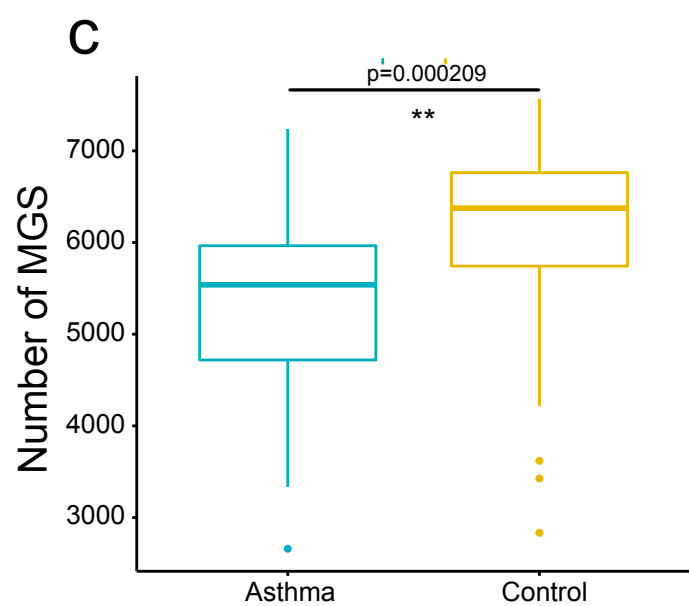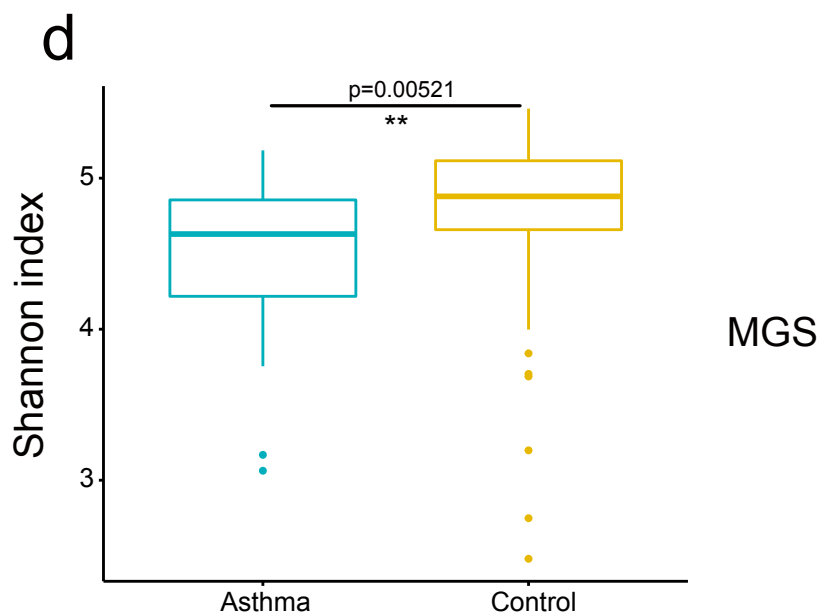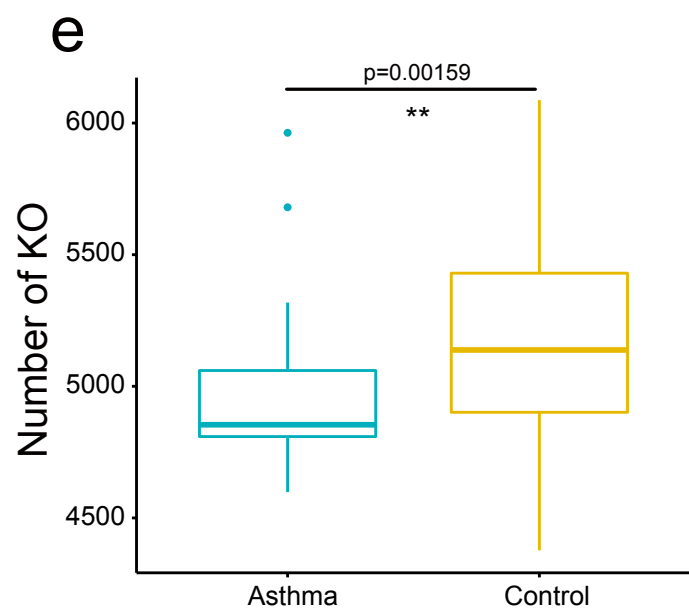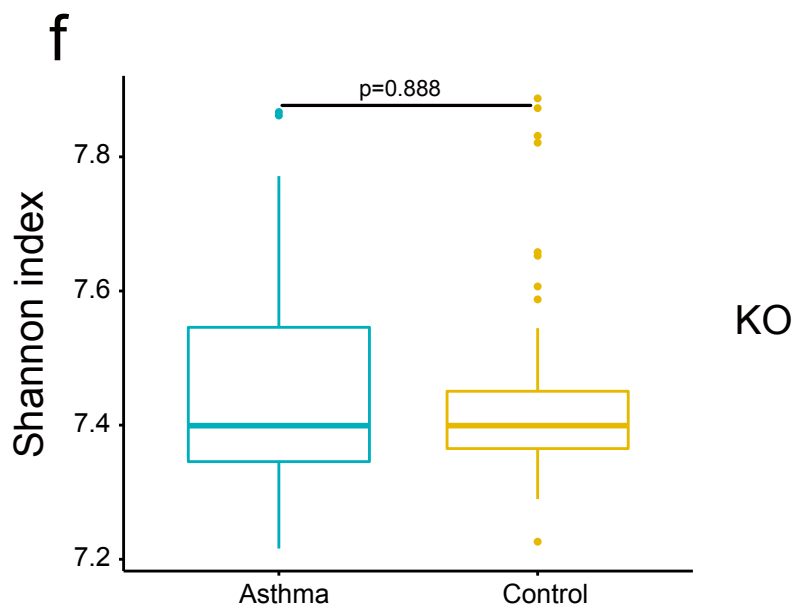

Supplement: Supplementary file 7 — Figure S4. Reduced gut microbial richness in only one twin sample. (a-f) Richness and alpha-diversity (Shannon index) at the gene, MGS and KO level of the two cohorts (Test by two-tailed Wilcoxon-rank sum test). Box plots showing both the richness values or diversity values and their density. (PDF 262 kb) [file 12866_2018_1257_MOESM7_ESM.pdf]

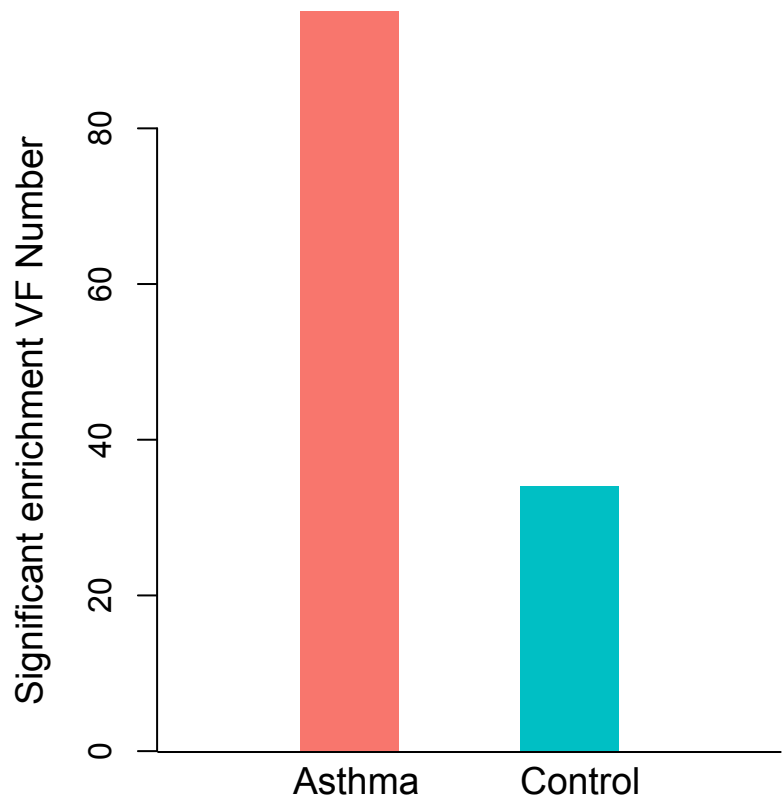

Supplement: Supplementary file 10 — Figure S3. The number of significant enrichment virulence factor (VF): we counted the significant (two-tailed Wilcoxon rank-sum test, P < 0.05) enrichment VF in different cohort. (PDF 92 kb) [file 12866_2018_1257_MOESM10_ESM.pdf]
